# Psychometric Properties of the Theory of Mind Task Battery (French Version) in Neurotypical Children and Intellectually Disabled Children

**DOI:** 10.3390/children11010079

**Published:** 2024-01-09

**Authors:** Nathalie Nader-Grosbois, Poline Simon, Emilie Jacobs, Marine Houssa

**Affiliations:** Psychological Sciences Research Institute, UCLouvain, 1348 Ottignies-Louvain-la-Neuve, Belgium; poline.simon@uclouvain.be (P.S.); emilie.jacobs@uclouvain.be (E.J.); marine.houssa@uclouvain.be (M.H.)

**Keywords:** Theory of Mind, empathy, emotion regulation, social skills, assessment, intellectual disability

## Abstract

These studies tested the psychometric properties of the French version of the Theory of Mind (ToM) Task Battery (vf) in typically developing (TD) children and intellectually disabled (ID) children. The Battery was administered to 649 TD children (2 ½ to 12 years old) in Study 1 and 155 ID (4 ½ to 14 ½ years old) in Study 2. Their mothers completed questionnaires: in both studies, the Theory of Mind Inventory (ToMI-1-vf); in Study 1, the Griffith Empathy Measure (GEM-vf) and the Emotion Regulation Checklist (ERC-vf); and in Study 2, the Social Competence and Behavior Evaluation (SCBE-vf). The Battery showed good internal consistency in both groups. Positive links with age and differences between age groups were identified in their performances. Convergent validity was confirmed by positive correlations between TD children’s scores in the Battery and in ToMI1-vf, in empathy, in emotion regulation, and by a negative correlation with emotion dysregulation. In ID children, their scores in the Battery were positively linked with those in ToMI-1-vf, in some scales of SCEB-vf, and had a low level of internalizing problems. This Battery presents good psychometric qualities and could be useful for explicit assessment of ToM in TD and ID children in future research and intervention.

## 1. Introduction

Given the growing interest in investigating new hypotheses about the development of emotional and social skills, notably abilities in Theory of Mind (ToM, originally defined by [1]), in neurotypical children and children presenting disorders, it is essential to use reliable and valid instruments that are easily administrable and adapted to them, from preschool to school level [2]. Assessment of children’s difficulties, weaknesses, and strengths in this regard is also important for effective prevention and clinical and psychoeducational intervention. More specifically, such measurement instruments will need to directly assess children’s profiles in the understanding of other people’s mental states, such as belief, knowledge, attention, pretense, intention, visual perspective, and thinking (cognitive ToM), as well as emotions and desires (affective ToM) (as explained by [3,4,5]), taking account of the development of these forms of ToM (from early ToM to first order or basic ToM to second order or advanced ToM) and of inter- and intra-individual variability. The literature has highlighted the considerable heterogeneity in ToM profiles and in the developmental trajectories of children presenting disabilities or neurodevelopmental disorders ([2]. The specific character of their ToM profiles and developmental progression must be examined, in clinical practice and in research projects, in connection with their skills or deficits in the cognitive, executive, communicative, emotional, affective, and social domains, taking account of individual factors (such as personality and type or severity of disorder) (e.g., [6,7,8]), and also of parental, family, and environmental factors (including education, special support, etc.). When the effectiveness of ToM training needs to be tested in experimental studies or in interventions, it is necessary to use validated ToM instruments as pre- and post-tests. Moreover, the inter-cultural comparison of ToM abilities in research requires common ToM measures, translated into different languages, to be available for the assessment of children from different countries.

Over the last few decades, various ToM measures have been devised, including observational measures, performance measures, and self-reported and hetero-reported questionnaires. In a meta-analysis, Beaudoin, Leblanc [9] listed 220 ToM measures for children, analyzed according to the types of measures (tasks or questionnaires), the mental states assessed, the methods of presentation or administration, the visual and audiovisual aids proposed, the number of items, the response methods, and the types of scores. Their psychometric properties are reviewed, and the typically and atypically developing populations to which these measures have been applied are mentioned. Some batteries offer several tasks assessing different levels of complexity of cognitive and/or affective ToM, for example, the Wellman & Liu Tests [10], the Animated Theory of Mind Inventory for Children (ATOMIC, [11]), the ToM-emotions and ToM-beliefs Tests [12], and the Theory of Mind Task Battery [13]; or its French version, [14]. These various tests and batteries are administered to children with autism spectrum disorder (ASD), behavior disorders (including attention deficit disorder with or without hyperactivity, ADHD), or intellectual disabilities (ID), from pre-school age to adolescence. Questionnaires, to be completed by parents or relatives, are also used, such as the ToM subscale of the Social Adaptation Scales for Children (EASE, [15]), the Theory of Mind Inventory (ToMI-1, [16]; or its French version, ToMI-1-vf, [17]), or the Empathy and Theory of Mind Scale (EToMs, [18]), assessing nice ToM, nasty ToM, and empathy in children aged between 3 and 5. These questionnaires cover pre-school age and sometimes school age. They have been used with children with various neurodevelopmental disorders, including ASD, especially the first version of ToMI. To assess the ToM of neurotypical (TD) and atypically developing children, it is useful to carry out both an assessment of the child’s performance in various tasks and an indirect assessment, consisting of a questionnaire filled out by an adult of his/her surroundings, such as a parent, a teacher, or another professional, on the basis of what they observe in the child’s daily life.

Given its interest, recently, researchers translated and adapted the ToM Battery, initially conceived in English, in other languages (e.g., Spanish, [19]; Tunisian Arabic, [20]; Chinese, [21]; Brazilian Portuguese, [22]; Hebrew, [23]; French, [16,24]). However, in these previous adaptation and validation studies, the sample sizes were often very limited, and children’s age ranges varied from one study to another. There is a lack of studies about this ToM Battery, involving large samples of children and targeting a great age range, for example, 12 years. A large body of literature has explored ToM skills in children with and without specific disorders. A number of these studies have used the ToM task Battery to assess skills. It should be noted that for some samples with specific disorders, only a few authors have chosen to administer this measure. Concretely, this ToM Battery has been administered to TD preschoolers [25,26] and children at school age (6–15 years, [24]; 7–14 years, [27]; 6–7 years, [28]; 7–12 years, [29]). Concerning its’ applicability, it has been administered to children presenting ASD [13,21,24,30,31,32,33,34,35,36,37,38], ADHD [33], externalized behavior disorders [39], ID [40,41], developmental language disorders [23], deaf children with spoken language multilingualism [42] and to patients with brain injury [22].

Some of these studies have investigated links between symptoms in these atypical and clinical groups of children and ToM abilities. Others have examined links between ToM (measured using the ToM Battery) and emotion regulation in TD children (e.g., [25]) or in ID children (e.g., [40]). The relationships between ToM, evaluated with the ToM Battery, and social skills have also been explored in TD children (e.g., [26]), in children with externalized behavior disorders [39], and in ID children (e.g., [41]). Links between executive functions and ToM, assessed by the ToM Battery, have also been investigated (e.g., [25,38]). The score in the ToM Battery has also been used to study the link between cerebral activity in emotion recognition and processing by children with autism spectrum disorder [31]. The ToM Battery has also been used as a pre- and post-test to assess the effectiveness of intervention (e.g., [20,25,39,40]). In developmental psychopathology and neuropsychology, as intricate links between ToM and empathy have been postulated, recent studies have begun the investigation of links between affective and cognitive ToM and the three dimensions of empathy. Affective empathy corresponds to an automatic emotional response and an ability to share other people’s emotions, while cognitive empathy refers to the capacity to adequately understand other people’s distress or emotions by decoding their socio-emotional cues and taking their perspective in social situations; behavioral empathy is shown through prosocial actions (e.g., [27,43,44,45,46,47]. In the literature, cognitive empathy and affective ToM are often considered quasi-synonymous by some authors (e.g., [44]). Recently, the ToM Battery has been used to examine relationships between ToM and empathy profiles in TD children [47].

For research purposes and to guide interventions toward children in preschool and school periods, a valid and reliable measure is required. It has to be based on a solid theoretical background and propose a performance assessment of explicit ToM that evaluates how children understand several affective and cognitive mental states in other people. Such a measure should provide an easy and quick assessment of the ToM strengths and weaknesses of children who live in different countries and cultures, including French-speaking children. As French is one of the five most widely spoken languages in the world, it is essential that researchers and psychologists have an adapted version of tools in French at their disposal. It is also relevant to check the applicability and psychometric properties of such a measure in French-speaking atypical populations, including children presenting intellectual disabilities who are at risk of delay or deficit in ToM skills and social maladjustment.

## 2. Objectives and Hypotheses of the Two Studies

These two studies aimed to check the psychometric properties of the French adaptation of the ToM Task Battery (ToM Battery-vf, [14]) in a great sample of typically developing (TD) children and in children with intellectual disabilities (ID), at preschool and school age, in view of its interest highlighted by the various ways in which it has been used in previous studies.

The first goal was to test the instrument’s reliability through its internal consistency. It was expected that, in terms of its total score and three subscores (affective, cognitive, and mixed ToM) the ToM Battery-vf would be found to have good or acceptable internal consistency in TD children (Study 1) and in ID children (Study 2).

The second aim was to examine whether these scores in ToM Battery-vf varied depending on age in TD children (Study 1) and in ID children (Study 2). It was expected that scores in TD children would be linked positively with age and differ by age group, reflecting an evolution of these abilities during the preschool and elementary school years. Moreover, Study 2 aimed to explore whether scores in the ToM Battery-vf varied depending on developmental age in ID children (Study 2); it was expected that their scores would be linked positively with their developmental age.

The third aim was to examine potential differences in the scores in ToM Battery-vf depending on gender. Based on an assumption of equivalence between girls and boys in French-speaking culture, it was predicted that scores in ToM Battery-vf would not differ depending on gender in TD children (Study 1) and in ID children (Study 2).

The fourth objective concerned construct validity by testing convergent validity through the links between scores in ToM Battery-vf and those in the French version of Theory of Mind Inventory-first version (ToMI-1-vf) in TD children (Study 1) and ID children (Study 2). It was hypothesized that positive associations would be found between explicit children’s performances in ToM Battery-vf and applied ToM skills as perceived by parents for TD children (Study 1) and ID children (Study 2).

A fifth objective was to explore the associations between scores in ToM Battery-vf and those in the Griffith Empathy Measure (GEM-vf) and the Emotion Regulation Checklist (ERC-vf) in TD children (Study 1). It was predicted that the ToM Battery-vf scores would be linked positively with empathy and emotion regulation, but negatively with emotion dysregulation. 

A final objective was to check associations between ToM Battery-vf scores and those in the Social Competence and Behavior Evaluation (SCBE), specifically in ID children (Study 2). It was predicted that significant correlations would be found between specific socio-affective dimensions or social competences in their profiles and some scores in ToM Battery-vf. It was further expected that some scores in ToM Battery-vf would correlate significantly with the risk of internalizing or externalizing problems in ID children.

## 3. Method

### 3.1. Participants

*In Study 1*, 649 children aged between 2 ½ years and 12 years and 4 months (*M* = 6 years and 2 months, SD = 31.10) participated. The sample consisted of 282 boys and 367 girls. The average family income corresponded to EUROS 3000–3500 to 3500–4000 a month. Most of the children were living with both parents. Children were excluded if they showed developmental delay or learning disabilities, checked by means of SON-R or some subscales of WPPSI-III [48] (e.g., matrix, reasoning, vocabulary, and information) administered by researchers. Concerning language skills, three criteria were applied. First, the native language had to be French in all cases; some children (*n* = 3) were excluded because their mother tongue was not French, and they had just begun their schooling in a French preschool class. This was confirmed by the teacher. Secondly, children had to be able to understand and respond to at least the first task of ToM Battery-vf. Third, children’s level of verbal intelligence capacity had to be at least equal to 2 ½ years. All of the TD preschoolers were recruited for the present study. Table 1 presents socio-demographic information about the levels of education of mothers and fathers, family income, and mode of care for children (including percentage distributions, or means and standard deviation). The level of education of parents was classified on a 7-point scale from low (elementary school not completed) to high (university or PhD). The average level of education was upper secondary school for mothers and a bachelor’s degree for fathers. The family’s monthly income was classified on a 12-point scale from low (EUROS 0–500) to high (EUROS 6000 or more).

*In Study 2*, 155 French-speaking children with non-specific intellectual disabilities or with trisomy 21 (T21, also known as Down Syndrome), aged between 4 years and 8 months and 14 years and 8 months (*M* = 9 years and 9 months, SD = 25.44), participated (100 boys and 55 girls). They all had a preschool developmental age, with a mean of 5 years and 1 month (SD = 13.90); 49% of these ID children were already included in a previous study [41]. Concerning inclusion and exclusion criteria, the children had to have been diagnosed as having a mild to moderate ID (IQ between 50 and 70), according to AAIDD (American Association on Intellectual and Developmental Disabilities, 2011) and DSM-V (the Diagnostic and Statistical Manual of Mental Disorders) criteria. Furthermore, children with specific genetic syndromes (such as Fragile-X or Williams) or an associated diagnosis of autism spectrum disorder were excluded. Children had to be between 4 and 14 years old and have a developmental age between 3 and 6 years; this was checked by means of SON-R or some subscales of WPPSI-III [48] (e.g., matrix, reasoning, vocabulary, and information) administered by researchers. ID children had to be able to understand and respond to at least the first task of ToM Battery-vf (corresponding to the emotion recognition task). Finally, about ID children, there was a preliminary check about their recent language assessment by a speech therapist (using ELO; [49]) or recent verbal intelligence testing (including vocabulary and information from the WPPSI-III [48]) by a psychologist. In the case of no recent assessment, the searchers checked whether ID children obtained higher levels of developmental ages in language abilities or verbal intelligence than 3-years (using ELO or WPPSI-III) to include them in the research. As shown in Table 1, the mean of parents’ level of education was 5.17 (SD = 2.71) for mothers and 5.13 (SD = 2.54) for fathers; 5 corresponded to 3 years of graduate school. The mean family’s monthly income was 4.45 (SD = 2.29); 4 corresponded to 1500–2000 and 5 corresponded to EUROS 2000–2500 a month. All participants were native French speakers.

### 3.2. Measures

#### 3.2.1. In Studies 1 and 2

*Theory of Mind Task Battery–French version* ([13,14]).

This Battery is designed to assess the ToM of young children of preschool age and older children whose cognitive and language skills are affected by a developmental delay or disorder, including autism spectrum disorders. Hutchins, Prelock [13] drew on the theoretical and empirical foundations to devise 9 tasks and 15 questions to assess these skills:

(1) Recognizing the facial expression of the four basic emotions (joy, anger, fear, and sadness) (questions 1 to 4).
(2) Taking the visual perspective of protagonists (questions 5 and 6).
(3) Inferring emotions based on a desire (question 7).
(4) Inferring beliefs based on perception (question 8).
(5) Inferring action based on perception (question 9).
(6) Understanding first-order false beliefs (question 10).
(7) Inferring emotions based on second-order beliefs (question 11), reality (question 12), and second-order emotions (question 13).
(8) Understanding the discrepancy between message and desire (question 14).
(9) Understanding second-order false beliefs (question 15).

During the test, the examiner reads short stories illustrated by images. These stories correspond to scenarios in which characters experience social situations that children may encounter in everyday life. To help the child infer the mental states of the characters, thought or speech bubbles are inserted into some of the images. In addition to questions relating to ToM skills, a number of ‘control’ questions check whether the child remembers the stages of the story. For each ToM question, Hutchins, Prelock [13] provided four possible answers: one correct answer and three incorrect answers. The tool also uses images to ensure that the child understands the questions and to make it easier for them to choose an answer from among the proposed answers by pointing to the corresponding image over and above their potential verbal response. The nine tasks, which are progressively complex, are divided into six first-order ToM tasks and four second-order ToM tasks. This child is asked fifteen questions, and his or her answers are scored (from 0 to 15 max.).

For the translation into French, the administration, scoring, and rating protocol of the ToM Battery were adapted [14]. A back-translation was performed by an expert, which was found to have a 99% degree of similarity with the original tool. Recently, a Manual of ToM Battery-vf [50] has been published, which summarizes the theoretical background, validation studies, and empirical studies that have used the tool to test hypotheses about typically and atypically developing children, administration, scoring, examples of clinical cases, and guidelines for clinical or psychoeducational intervention, individually and in groups.

The Battery allows calculating three specific scores (corresponding to three factors) for “affective ToM”, “cognitive ToM” and “mixed ToM”. The “affective ToM” score corresponds to the recognition of the four basic emotions, desire and attention (questions 1, 2, 3, 4, 7, and 14; maximum 6 points). The “cognitive ToM” score corresponds to the ability to infer knowledge about the other person based on what they see, to take the other person’s visual perspective, and to understand the links between actions and perception, beliefs, and false beliefs (questions 5, 6, 8, 9, 10, and 15; maximum 6 points). The “mixed ToM” score corresponds to the inference of emotion-inducing beliefs, emotions in the context of real events, and thoughts about other people’s emotions (questions 11, 12, and 13; maximum 3 points). Points for the questions relating to each component must be added together to calculate specific scores for the mental states concerned. The psychometric qualities of the ToM Battery-vf were tested in two validation studies about preschoolers and children at the beginning of elementary school [14]. In the first validation study, the performance in the ToM Battery-vf of 209 Belgian children aged between 3 and 6 ranged from 3 to 14 (*M* = 8.39; SD = 2.34). Internal consistency was satisfactory, as measured by Cronbach’s alpha (α = 0.75). Principal component factor analysis with Varimax rotation resulted in a progressive three-factor structure for “emotion recognition” (α = 0.741), “perspective taking” (α = 0.736), and “complex inference” (α = 0.775). The ToM Battery-vf score varied according to age, presenting a highly significant positive correlation (*r* = 0.64, *p* < 0.001) and significant differences between the age groups (3, 4, 5, and 6 years), as measured by the ANOVA (*F* (3, 207) = 8.794, *p* < 0.001) and Bonferroni multiple comparisons. In terms of external validity, in the first study, the correlations were positive and highly significant between the ToM Battery-vf score and scores in the ToM-emotions and beliefs tests [12] and those in the questionnaires ToMI-1-vf [17] and EASE-ToM and social skills [29].

In the second study, conducted on a subsample of 82 Belgian children aged from 3 to 6 years, correlations were positive and highly significant for test-retest reliability (*r* = 0.87, *p* < 0.001) and inter-rater agreement (*r* = 0.97, *p* < 0.001; 99% agreement). Moreover, the children’s ToM Battery-vf score at time 1 correlated positively and very significantly with their scores in the ToM-emotions and ToM-beliefs tests, in the ToMI-1-vf, in EASE (ToM and social skills), and in some scales of the Social Competence and Behavior Evaluation (SCBE; [51]) relating to the prosocial and cooperative dimensions and general adaptation.

*Theory of Mind Inventory-1–French version* (*ToMI-1*, [52]; *ToMI-1-vf*, [17]).

This questionnaire, which was completed by mothers in the study, evaluates caregivers’ perceptions of the understanding of the mental states of children from 2 to 12 years old. The ToMI-1-vf is composed of 39 items assessing emotions, beliefs, desires, intentions, or perceptions. The French version matched the validation of the original version. Test-retest reliability (*r* = 0.86, *p* < 0.001) and internal consistency (α = 0.94) are very significant [17]. A manual of ToMI-1-vf [53] explains the theoretical background, the scoring, uses in research, and examples of clinical cases and guidelines for intervention.

#### 3.2.2. In Study 1

*Griffith Empathy Measure–French version* (*GEM*, [54]; *GEM-vf*, [47]).

Mothers also completed the French version of the Griffith Empathy Measure (GEM, [54]; GEM-vf, [47]), which assesses their perception of children’s affective (13 items) and cognitive empathy (4 items). Through 17 items, mothers indicate their degree of agreement on a 9-point Likert scale, ranging from “strongly disagree” (−4) to “strongly agree” (4). For each subscale, a mean score from −4 to 4 points is computed. The GEM was designed to evaluate the skills of children and adolescents aged between 4 and 16 years, whereas the French version, GEM-vf, has been validated among children aged 3 to 12 years. The internal consistency of GEM-vf was found on the basis of values of Cronbach’s alpha to be good for affective empathy (α = 0.83) but limited for cognitive empathy (α = 0.62). It was acceptable for total empathy (α = 0.79). The validation study confirmed good psychometric properties [47].

*Emotion Regulation Checklist–French version* (*ERC*, [37]; *ERC-vf*, [55]).

This questionnaire, which was completed by mothers of TD children, evaluates their perception of children’s ER abilities in daily life through 24 items. The ERC consists of two scales: emotion regulation and emotion dysregulation. The French validation of the ERC matched that of the original version and showed good internal consistency, with a Cronbach’s alpha for the emotion dysregulation subscale of 0.82 and for the emotion regulation subscale of 0.72. The correlation between these two scales is negative and significant (*r* = −0.66, *p* < 0.001). The validation study, or ERC-vf, confirmed good psychometric properties [55].

#### 3.2.3. In Study 2

*Snijders-Oomen-Revised (SON-R*, [56]).

This non-verbal scale is used to estimate the developmental age of ID children. Through six subscales, children’s performance and reasoning skills are assessed. This evaluation was used to ensure that children had a developmental age of 3 to 6 years to meet the criteria for inclusion.

*The Social Competence and Behavior Evaluation* (*SCBE*, [51]).

The SCBE assesses the social and affective abilities required for social adjustment. The questionnaire includes 80 items divided into eight basic subscales: (1) depressive-happy, (2) anxious-secure, (3) isolated-integrated, (4) dependent-autonomous, (5) angry-tolerant, (6) aggressive-controlled, (7) egoistic-prosocial, and (8) resistant-cooperative. These are used to form four global SCBE components: social competence, internalizing problems, externalizing problems, and general adaptation (all basic scales). For each subscale and global component, the higher the score, the fewer behavioral/affective difficulties the child has. The French version of the SCBE was validated on a sample of 800 preschoolers and demonstrated good properties, with a high inter-judge agreement, high internal consistency, good test-retest correlations, and no correlation with social desirability. This questionnaire was administered in Study 2.

### 3.3. Procedure

*For Study 1*, the Ethics Committee of the Research Institute in Psychological Sciences approved the research procedure of the study (Projet2020-40). Participants were recruited in French-speaking Belgian schools. TD children were between 2 ½ and 12 ½ years old. Children who had behavior problems, language disorders, developmental delays, intellectual disabilities, or autism symptoms were excluded. Information letters and consent forms were sent to parents. Children were tested by means of ToM Task Battery-vf individually at school in a quiet room. Parents completed the socio-demographic questionnaire. Mothers completed the ToMI-1-vf, the GEM-vf, and the ERC-vf.

*For Study 2*, the research procedure of the study (Projet2015-42; Projet2019-B403201941776) was also approved by the Ethics Committee of the Research Institute in Psychological Sciences Hospital-Faculty Ethics Committee of Saint-Luc UCLouvain. Children and their parents were recruited via Belgian specialized schools for children with mild to moderate intellectual disability or via an association of parents of children with Trisomy 21. The evaluation took place in a quiet room at the child’s school or at home and was completed during two sessions lasting 30–45 min. The children were assessed by means of the SON-R to estimate their developmental age. They then took the ToM Battery-vf. Parents completed the French version of the Theory of Mind Inventory-1 (ToMI-1-vf) and the French version of the Social Competence and Behavior Evaluation (SCBE). Parents could choose whether to complete these questionnaires at home or to make an appointment with the researcher to complete them with her help, if necessary.

## 4. Results

### 4.1. Descriptive Statistics

*For Study 1*, about the TD children, ToM Battery-vf scores ranged from 0 to 15 (*M* = 9.13; SD = 3.48). Table 2 presents descriptive statistics for ToM Battery-vf scores and for ToMI1-vf, GEM-vf, and ERC-vf, for the whole sample, for girls and boys, and for the different age groups in the TD sample. The *t*-test results indicated that there was no significant difference depending on gender in performance in ToM Battery-vf, despite a difference in mothers’ perception of their children’s skills in ToM in daily life (in ToMI1-vf) in favor of girls. A significant difference was found in emotion dysregulation to the disadvantage of boys, but not in emotion regulation. No significant difference in empathy components was found between girls and boys.

*For Study 2*, about the sample of ID children, the ToM Battery-vf scores ranged from 2 to 14 (*M* = 8.25; SD = 2.43).

Table 3 presents descriptive statistics for the entire sample and separately for boys and girls for scores in ToM Battery-vf, in ToMI-1-vf, and in SCBE in the ID sample.

The *t*-test results indicated that there was no significant difference depending on gender in scores in ToM Battery-vf and in ToMI-1-vf in this ID sample. In terms of the socio-affective profiles in SCBE, there were significant differences between girls and boys for isolated-integrated, angry-tolerant, aggressive-controlled, egoistic-prosocial, externalized problems, general adaptation, and interactions with peers. Each difference was in favor of girls (girls were described as more tolerant, controlled, and prosocial than boys, but also as having fewer externalized problems, better interactions with peers, and better general adaptation than boys), except for the subscale ‘isolated-integrated’ (boys were considered more integrated than girls).

### 4.2. Internal Consistency

*In Study 1*, for the TD sample, the internal consistency of the ToM Battery-vf, assessed by applying Cronbach’s alpha, was high (α = 0.83). The inter-item correlations were positive and significant (from 0.10 to 0.85, *p* < 0.02 to *p* < 0.00). In terms of the internal consistency for each factor of the ToM Battery-vf, the values of Cronbach’s alpha varied: α = 0.68 for the affective score, α = 0.67 for the cognitive score, and α = 0.88 for the mixed score.

*In Study 2*, for the ID sample, the internal consistency of the total ToM Battery-vf, assessed by applying Cronbach’s alpha (α = 0.67), was nearly acceptable. The inter-item correlations were positive and significant (from 0.17 to 0.75, *p* < 0.05 to *p* < 0.001).

### 4.3. Study 1: Progression Depending on Age, Differential Content Validity, and Percentiles

**Figure 1:**
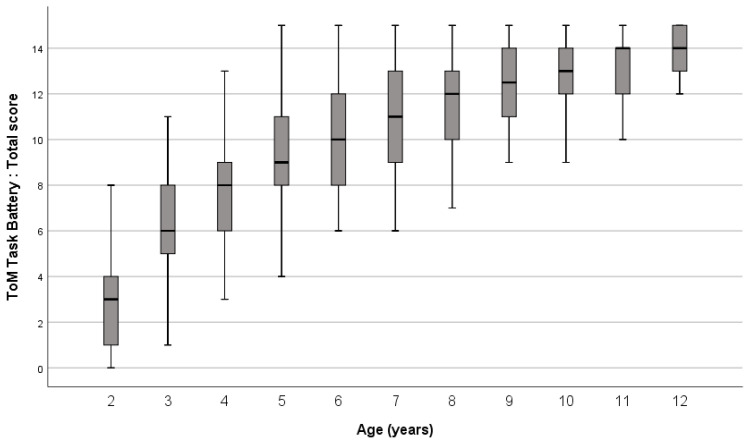
Distribution of ToM Battery-vf total scores by age (at one-year intervals) in TD children.

Two initial analyses (correlational and age group comparison) showed a progression of TD children’s scores in ToM Battery-vf by age (represented in Figure 1). First, a positive and significant correlation was obtained between the ToM Battery-vf total score and children’s age (*r* = 0.738, *p* < 0.001). Children’s age also correlated positively and highly significantly with subscores of the ToM Battery-vf: *r* = 0.53 for the affective score, *r* = 0.72 for the cognitive score, and *r* = 0.51 for the mixed score (*p* < 0.001). Second, children were divided into four age groups: Group 1 from 2 to 3 years (*n* = 143); Group 2 from 4 to 6 years (*n* = 322); Group 3 from 7 to 9 years (*n* = 117); and Group 4 from 10 to 12 years (*n* = 88). Table 2 presents the mean scores and standard deviations for each age group for all measures. A one-way ANOVA indicated a significant difference between these age groups in ToM Battery-vf, *F*(3) = 250, *p* < 0.001. Post-hoc Bonferroni confirmed significant differences between age groups (*p* < 0.001). Figure 1 represents the progression of scores year by year.

Subscale and composite cut-off scores were identified by age at intervals of 1 year (see Table 4). Similarly, in the validation study of the original version of this Battery, two levels of percentiles are provided (the 10th and 15th percentiles). The results of this analysis could be useful in indicating whether an individual, depending on his or her age, could be at risk of poor explicit ToM skills, in terms of the total score and in the three specific scores for affective ToM, cognitive ToM, and mixed ToM.

The comparisons between age groups for other measures (see Table 3) also showed significant differences in scores of ToMI-1-vf, emotion regulation, and empathy.

### 4.4. Study 2: Progression and Links between ToM Battery-vf Scores and Chronological or Developmental Age

**Figure 2:**
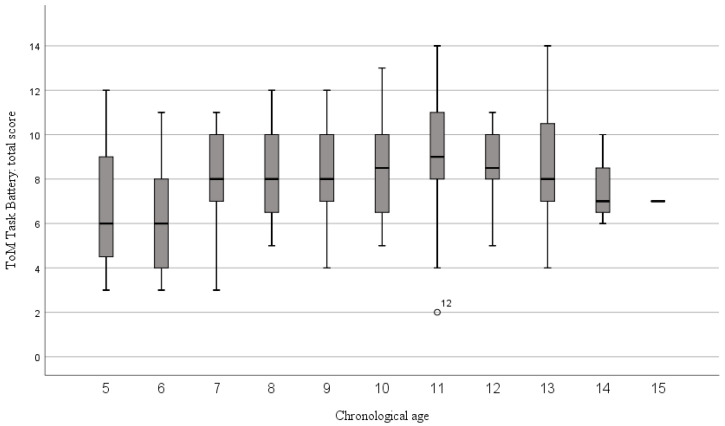
Distribution of ToM Battery-vf total scores by chronological age (in one-year intervals) in ID children.

**Figure 3:**
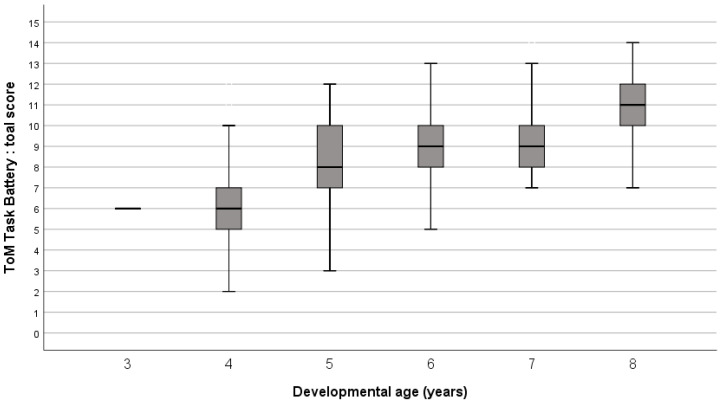
Distribution of ToM Battery-vf total scores by developmental age (in one-year intervals) in ID children.

Positive and significant correlations were obtained between the total scores in ToM Battery-vf and in ToMI-1-vf attained by ID children and, respectively, their chronological age and developmental age. Chronological age and developmental age, respectively, were also correlated with the affective score of ToM Battery-vf. However, only developmental age correlated positively and significantly with cognitive and mixed scores (see Table 5). The progression of total score in ToM Battery-vf is represented year by year, for chronological age in Figure 2, and for developmental age in Figure 3.

### 4.5. Convergent and Concurrent Validity

*In Study 1*, the external validity of the ToM Battery-vf was verified with reference to questionnaires, the ToMI-1-vf, the GEM-vf, and the ERC-vf, by calculating Pearson’s correlation coefficients (see Table 6). In addition, we carried out partial correlation analyses while controlling for age in this TD group. The partial correlations stayed significant even when controlling for age between ToM task Battery-vf total score and ToMI scores (*r =* 0.19; *p* = 0.002), and between ToM task Battery-vf total score and ERC scores (emotion regulation score, *r =* 0.17; *p* = 0.005; emotion dysregulation score, *r =* −0.13; *p* = 0.026). However, between ToM task Battery-vf total score and GEM scores, the partial correlations are no longer significant when age is controlled. In other instrumental studies about this Battery, none of the authors tested its convergent and concurrent validity in controlling for age (e.g., [14,19,20,21,22,23,50]); therefore, we did not control age.

As expected, correlations were positive and significant between the total score and the three subscores of ToM Battery-vf on the one hand, and the total score in ToMI1-vf, the three scores in empathy, and the score in emotion regulation on the other hand. Furthermore, the correlation between the total score and the three subscores of the ToM Battery-vf and the emotion dysregulation score was negative and significant.

*Regarding Study 2*, as shown in Table 7, the total score and specific scores in affective and cognitive ToM obtained by ID children were linked positively and significantly with the total score in ToMI-1-vf based on the mother’s perception. Only the mixed score did not correlate with the ToMI-1-vf. When controlling age, the partial correlations stayed significant between ToMI-1-vf and the total score of ToM Battery-vf (*r* = 0.30; *p* = 0.038), as well as the cognitive score of the Battery (*r* = 0.31; *p* = 0.033). When controlling for developmental age, the partial correlations were not significant.

Always for the sample of ID children, the total score in ToM Battery-vf correlated positively with those in the SCBE subscales depressive-happy (*r* = 0.18, *p* < 0.05; *r* = 0.21, *p* < 0.02 when controlling for chronological age), isolated-integrated (*r* = 0.18, *p* < 0.05; *r* = 0.21, *p* = 0.02 when controlling for chronological age), and internalizing problems (*r* = 0.16, *p* < 0.05; *r* = 0.23, *p* = 0.01 when controlling for chronological age). Furthermore, when controlling the chronological age, the total score in ToM Battery-vf correlated with the subscale dependent-autonomous (*r* = 0.18; *p* = 0.04). Finally, when partial correlations were performed with developmental age, no significant correlation was obtained.

Their affective score in ToM Battery-vf correlated positively with those in the SCBE subscales depressive-happy (*r* = 0.17, *p* < 0.05), anxious-secure (*r* = 0.16, *p* < 0.05), isolated-integrated (*r* = 0.19, *p* < 0.05), social competences (*r* = 0.21, *p* < 0.005), and general adaptation (*r* = 0.17, *p* < 0.05).

## 5. Discussion

The studies aimed to validate the ToM Battery-vf and assess its psychometric properties in TD children aged between 3 and 12 years (Study 1) and in children with ID functioning at a developmental age equivalent to 3 to 6 years (Study 2).

First, as was expected, the internal consistency was good for the total score and the mixed score of ToM Battery-vf in TD children from 3 to 12 years old and acceptable for the two subscores (affective and cognitive) (Study 1). For ID children, internal consistency was acceptable for the mixed score, but lower than initially expected for the other scores (Study 2). Future studies should test internal consistency for total and specific scores with a larger sample of ID children, as few studies have used this Battery with this population group.

Second, as was expected, the scores (total, affective, cognitive, mixed) in ToM Battery-vf correlated positively and highly significantly with age and differed significantly between age groups (2–3, 4–6, 7–9, 10–12 years), suggesting a progression in the explicit ToM skills at preschool and elementary school age in TD children (Study 1). They move from early, to first-order or basic, and to second-order or advanced ToM capabilities, depending on their age. These findings are consistent with those obtained by previous studies reporting a progression with age in terms of total score and affective ToM and cognitive ToM scores in this Battery [20,24].

For ID children functioning cognitively at a level equivalent to a developmental age of between 3 and 6 years, positive and significant correlations were obtained between both the total score and affective score in ToM Battery-vf and both their chronological age and developmental age, respectively. However, only their developmental age correlated with the cognitive and mixed scores. The older ID children were, and the higher their developmental age was, the more they were able to solve ToM tasks, particularly those requiring the understanding of affective mental states. As ID children attained a higher developmental age, they grew more successful at tasks requiring the understanding of cognitive mental states. These findings are in line with those obtained by Jacobs, Simon [41].

The calibration of the ToM Battery-vf makes it possible to specify the respective percentiles to which the child’s abilities correspond for the total score as well as the scores for the subscales. The percentiles provide a type of ordinal-level normative score and can facilitate interpretation as they indicate the percentage of children in the ‘normative’ study sample whose scores are equal to or below a given score, bearing in mind that these percentiles are determined by the nature of the underlying distribution of scores. In line with the results for the original version [32], it is interesting to use the reference to 10th or 15th percentiles identified in TD children (Study 1) to compare the score obtained in ToM Battery-vf by individual ID children, according to their chronological age, in order to estimate the delay in their explicit ToM, or according to their developmental age, to assess whether there was a major deficit in ToM, despite a potentially similar cognitive level to TD children presenting the same developmental age.

Third, based on an assumption of equivalence between girls and boys in French-speaking culture, it was predicted that scores in ToM Battery-vf would not differ depending on gender. As expected, no significant difference depending on gender in ToM Battery-vf scores was found in either TD children or ID children, although some gender differences were found for emotion dysregulation in TD children and for specific socio-affective dimensions between ID girls and ID boys. No previous studies of ID children have examined a possible gender effect on performance in this Battery.

Fourth, construct validity was verified by testing convergent validity through associations between scores in ToM Battery-vf and those in ToMI-1-vf, GEM-vf, and ERC-vf for TD children and in ToMI-1-vf and SCBE for ID children. As was hypothesized, in TD children, positive links were perceived by their mothers, who had observed them in daily life, between their explicit performance in ToM Battery-vf and their applied ToM skills, empathy abilities, and emotion regulation. As expected, negative and significant links were obtained between these ToM Battery-vf scores and emotion dysregulation in TD children. Moreover, as these children attain higher levels of explicit ToM and affective and cognitive empathy, they experience less difficulty regulating their own emotions during social interactions with peers and adults and in frustrating situations. These significant associations support the convergent validity of ToM Battery-vf in TD children aged 2 to 12 years. These findings are consistent with the positive links found between scores in this Battery and in ToMI in other studies [14,32], even when controlling age. Positive links between scores in this Battery and emotion regulation and negative links with emotion dysregulation have also been emphasized in previous studies of TD children (e.g., [25]).

In ID children, specific scores in affective and cognitive explicit ToM in the ToM Battery-vf were found to be positively and significantly linked with the total score in ToMI1-vf based on the mothers’ perception. Only the mixed score of the Battery did not correlate with the ToMI1-vf. Thus, the better ID children were at solving tasks requiring the understanding of affective and cognitive mental states, the more their mothers reported higher abilities in applying ToM in daily life, and inversely. Some previous studies have also reported a positive association between the ToM Battery and the ToMI in ID children (e.g., [40]) and in children with autism spectrum disorders (first version, [36]; or second version, [21,35]). Moreover, in ID children, significant links have been found between some ToM Battery-vf scores and some scores in SCBE. Similar results were found when chronological age was controlled. An additional link is highlighted when chronological age is controlled; indeed, the more autonomous children are perceived to be, the better their ToM skills. For this ID sample, the total score in explicit ToM in ToM Battery-vf correlated positively with the socio-affective dimensions of depressive-happy, isolated-integrated, and with a low level of internalizing problems. ID children who were better at ToM tasks assessing their understanding of diverse mental states were more likely to come across as integrated in interactions with their peers and to appear happy, and they were less likely to present internalizing problems. In particular, ID children who achieved higher affective scores in this Battery, thus showing a better understanding of affective mental states, also scored higher for social competences and general adaptation and came across as integrated, happy, and secure in their interactions with peers or adults. Along the same lines, positive links between scores in ToM Battery-vf and social adjustment in ID children have been found previously by Jacobs, Simon [41]. These findings of significant associations also confirmed the convergent validity of ToM Battery-vf for ID children aged between 4 and 14 years and with a developmental age equivalent to 3 to 6 years.

Concerning the difference between TD and ID children, while for TD children the development of ToM competences follows steps depending on age, for ID children there is a developmental delay in cognitive ToM attesting by literature [41,57,58,59]. The present study confirms this result. The ToM Task Battery allowed for identifying delays or deficits in cognitive ToM. Regarding the development of affective ToM in previous studies [29,41], the present findings highlight the importance of considering developmental age and chronological age for ID children. Social experience plays a role in the development of affective ToM. Moreover, the more ID children have competences in affective ToM, the more they are perceived as socially competent [41]. Also, explicit and applied ToM seem to be in line for both TD and ID children. In fact, the more children have ToM competence, the more their mothers report higher ToM competences in daily life.

To conclude, the ToM Battery–French version presents good psychometric properties. However, some limitations of the two present studies must be pointed out: there were no other performance measures of ToM and no systematic assessment of language skills, and divergent validity was not tested. Future research could continue to check convergent validity by using another direct performance measure of ToM (not only questionnaires as in these two studies). In view of the potential limitation of applicability to children with a low level of language comprehension and reception, further research should more specifically explore links between ToM performance in this Battery and children’s language level in different dimensions (pragmatic, semantic, syntactical, lexical, comprehension, and reception). It could be interesting to collect data on ToM and language skills as expressed by children in spontaneous narrative in the test “La Chute dans la Boue” (a subtest of Nouvelles Epreuves pour l’Examen du Langage, N-EEL, [60]) in order to investigate how these abilities are linked with their performance in this ToM Battery-vf. Finally, it could be useful in both a research and an intervention context to combine the use of the ToM Battery and the Theory of Mind Inventory (ToMI-vf, first version [17] or second version [61]), which are designed with reference to a common theoretical foundation. This would make it possible to gather information from different sources in order to establish an individual profile of strengths and weaknesses in the understanding of affective and cognitive mental states from the time of its emergence through to the development of first-order and advanced second-order ToM. Future research could explore cross-sectional and longitudinal studies in which explicit and applied ToM, assessed by these two tools, are linked with affective, cognitive, and behavioral empathy in TD children, in ID children, or in children presenting ASD and ADHD, externalized and internalized behavior disorders [62]. In these atypically developing children, links between “hot” and “cold” executive functions and affective or cognitive ToM skills should be examined in depth, as made in a study about ASD children led by Yu, Li [38]. These questions could be examined in comparing clinical groups and in identifying clinical subgroups (notably by applying hierarchical cluster analyses of cases) according to their ToM profiles and their levels in these other domains. Future studies could investigate inter-cultural comparisons of ToM development in children who speak different languages, who benefit from various qualities of social interactions, of language family environment at home, and of parent-child conversations of emotions and mental states.

## Figures and Tables

**Table 1:** Demographic characteristics of participants’ families for Studies 1 and 2.

	TD Children		ID Children	
	*M* (SD)	%	*M* (SD)	%
*Mothers’ education level*	4.99 (1.16)		5.17 (2.71)	
Primary school not completed		0.9%		9.8%
Primary school		1.5%		19.5%
Lower secondary school		9.8%		23.1%
Upper secondary school		16.5%		13.4%
Bachelor’s degree		30.7%		17.1%
Master’s degree		37.5%		14.6%
PhD		3%		2.4%
*Fathers’ education level*	4.74 (1.24)		5.13 (2.54)	
Primary school not completed		1%		5.9%
Primary school		1.8%		14.7%
Lower secondary school		17%		38.3%
Upper secondary school		22.6%		20.6%
Bachelor’s degree		21.1%		8.9%
Master’s degree		34.7%		11.8%
PhD		1.9%		5.9%
*Family income*	7.85 (2.62)		4.45 (2.29)	
0–499		0.2%		2.1%
500–999		0.8%		7.3%
1000–1499		5.6%		34.4%
1500–1999		6.6%		24%
2000–2499		5.8%		11.5%
2500–2999		11.1%		2.1%
3000–3499 3500–3999		8.9% 12.1%		3.1% 4.2%
4000–4499		27%		10.4%
4500–4999		8.2%		1%
5000–5499		4.9%		0.0%
5500–5999		3.3%		1%
6000 or more		5.3%		0.0%
*Mode of care*				
Child lives with both parents together		83.1%		50.9%
Child lives in an alternating custody arrangement		6%		12%
Child lives only or principally with their mother		7.3%		16.7%
Child lives only or principally with their father		3%		0.9%
Others				19.4%

Note: *M* = mean; SD = standard deviation; TD = typically developing; ID = intellectually disabled.

**Table 2:** Descriptive statistics for TD children and between-age group and gender comparisons.

		By Gender			By Age Group				
	Total	Girls	Boys		Group 1 2 to 3 yo	Group 2 4 to 6 yo	Group 3 7 to 9 yo	Group 4 10 to 12 yo	
N	649	367	282		143	322	117	88	
	*M* (SD)	*M* (SD)	*M* (SD)	*t*	*M* (SD)	*M* (SD)	*M* (SD)	*M* (SD)	*F*
ToM Battery-vf total (max = 15)	9.13 (3.48)	9.27 (3.44)	8.94 (3.52)	1.19	5.00 (2.64)	9.01 (2.40)	11.43 (2.36)	13.02 (1.76)	250.02
ToM Battery-vf–Affective (max = 6)	5.06 (1.26)	5.10 (1.21)	5.00 (1.31)	0.95	3.56 (1.58)	5.29 (0.85)	5.63 (0.64)	5.77 (0.48)	138.60
ToM Battery-vf–Cognitive (max = 6)	2.89 (1.77)	2.89 (1.77)	2.88 (1.78)	0.10	1.12 (1.17)	2.66 (1.36)	4.04 (1.38)	4.98 (1.03)	196.13
ToM Battery-vf–Mixed (max = 3)	1.19 (1.29)	1.27 (1.31)	1.08 (1.26)	1.85	0.31 (0.69)	1.08 (1.18)	1.71 (1.37)	2.30 (1.19)	63.84
ToMI1-vf score (max = 20)	14.99 (2.76)	15.30 (2.66)	14.61 (2.82)	2.76 **	12.11 (2.59)	15.08 (1.81)	16.91 (2.04)	17.54 (1.89)	130.17
GEM-vf total (max = 17)	1.24 (1.01)	1.26 (1.04)	1.22 (0.97)	0.36	0.78 (0.78)	1.18 (0.96)	1.67 (1.04)	1.67 (1.10)	16.50
GEM-vf–Affective (max = 13)	1.35 (1.09)	1.41 (1.09)	1.29 (1.09)	1.14	0.88 (0.96)	1.31 (1.01)	1.84 (1.11)	1.68 (1.21)	13.53
GEM-vf–Cognitive (max = 4)	1.13 (1.50)	1.11 (1.53)	1.16 (1.47)	−0.34	0.69 (1.24)	1.05 (1.48)	1.51 (1.40)	1.66 (1.77)	7.45
ERC-vf–regulation (max = 4)	3.29 (0.40)	3.26 (0.41)	3.33 (0.37)	−1.48	3.23 (0.37)	3.33 (0.38)	2.54 (1.37)	3.12 (0)	5.52
ERC-vf–dysregulation (max = 4)	1.96 (0.40)	1.93 (0.38)	2.01 (0.42)	−1.91 *	2.08 (0.39)	1.92 (0.39)	1.27 (.44)	1.67 (0)	7.10

Notes. * *p* < 0.05; ** *p* < 0.01; ToM = Theory of Mind; ToMI1-vf = Theory of Mind Inventory 1–French version; GEM-vf: Griffith Empathy Measure–French version; ERC-vf = Emotion Regulation Checklist–French version; *M* = mean; SD = standard deviation.

**Table 3:** Descriptive statistics for ID children and differences between boys and girls.

ID Children			Total	Girls	Boys	
	Min	Max	*M* (SD)	*M* (SD)	*M* (SD)	*t*
ToM Battery-vf total score (max = 15)	2	14	8.25 (2.43)	8 (2.21)	8.38 (2.55)	−0.93
ToM Battery-vf–Affective score (max = 6)	2	6	5.08 (1.05)	5.07 (1.09)	5.09 (1.04)	−0.08
ToM Battery-vf–Cognitive score (max = 6)	0	6	2.52 (1.32)	2.43 (1.15)	2.57 (1.41)	−0.64
ToM Battery-vf–Mixed score (max = 3)	0	3	0.64 (0.93)	0.5 (0.87)	0.72 (0.96)	−1.39
ToMI1-vf total score (max = 20)	8.11	19.73	13.27 (2.97)	12.76 (3.22)	13.53 (2.85)	−1.23
SCBE–depressive-happy (max = 50)	14	50	35.66 (6.64)	36.19 (5.87)	35.37 (7.04)	0.73
SCBE–anxious-secure (max = 50)	11	50	32.32 (7.47)	32.50 (7.61)	32.22 (7.43)	0.22
SCBE–isolated-integrated (max = 50)	10	48	34.62 (6.91)	34.59 (6.52)	34.63 (7.16)	3.53 *
SCBE–dependent-autonomous (max = 50)	7	46.67	28.71 (7.32)	28.16 (6.66)	29.01 (7.68)	−0.69
SCBE–angry-tolerant (max = 50)	3	44	26.71 (7.76)	29.57 (7.04)	25.13 (7.72)	3.53 ****
SCBE–aggressive-controlled (max = 50)	10	44	31.79 (6.06)	34.04 (4.80)	30.36 (6.22)	4.18 ****
SCBE–egoistic-prosocial (max = 50)	7	47	28.25 (7.61)	30.94 (6.18)	26.77 (7.94)	3.38 *
SCBE–resistant-cooperative (max = 50)	10	50	33.43 (7.48)	34.64 (6.76)	32.77 (7.80)	1.50
SCBE–social competences (max = 200)	49	174.50	113.07 (25.23)	117.02 (22.73)	110.89 (26.37)	1.45
SCBE–internalizing problems (max = 100)	23	93	70.33 (12.75)	70.56 (10.39)	70.20 (13.93)	0.17
SCBE–externalizing problems (max = 100)	26	100	68.10 (14.68)	73.43 (13.15)	65.17 (14.72)	3.47 ***
SCBE–general adaptation (max = 400)	125	359	251.50 (41.70)	261.01 (35.91)	246.27 (43.85)	2.13 ***
SCBE–interactions with peers (max = 50)	15	45	31.54 (5.37)	33.31 (4.55)	30.59 (5.56)	3.11 *
SCBE–interactions with adults (max = 50)	12.38	44	31.07 (6.31)	31.40 (5.84)	30.89 (6.58)	0.48

Notes. * *p* < 0.05; *** *p* < 0.005; **** *p* < 0.001; Min = minimum; Max = maximum; M = mean; SD = standard deviation. ToM = Theory of Mind; ToMI1-vf = Theory of Mind Inventory 1–French version; SCBE = Social Competence and Behavior Evaluation; ID = intellectually disabled.

**Table 4:** Percentiles by age for scores of ToM Battery-vf in TD children.

Age (Years)	*n*	Total Score	
		10th %ile	15th %ile
2	47	1	1
3	89	3	3
4	103	5	6
5	144	6	7
6	71	7	8
7	44	7	8
8	37	8	9
9	35	10	10
10	48	10	10
11	29	11	11
12	9	12	12

Note: *n* = number of children; %ile: percentile.

**Table 5:** Correlations between chronological age, developmental age, and scores in ToM Battery-vf and ToMI1-vf for ID children.

	Total ToM Battery-vf	Affective Score	Cognitive Score	Mixed Score	ToMI1-vf
Chronological age	0.20 *	0.21 *	0.14	0.09	0.25 *
Developmental age	0.57 **	0.59 **	0.35 **	0.32 **	0.32 **

Notes. * *p* < 0.05; ** *p* < 0.01; ID = intellectually disabled.

**Table 6:** Correlations between scores in ToM Battery-vf, ToMI1-vf, GEM-vf, and ERC-vf in TD children.

	ToM Battery-vf				ToMI1-vf	GEM-vf			ERC-vf	
	Total	Affective	Cognitive	Mixed		Total	Affective	Cognitive	RE	Dys
ToM Battery-vf total		0.76 ****	0.87 ****	0.76 ****	0.64 ****	0.26 ****	0.233 ****	0.185 ****	0.15 *	−0.23 ****
ToM Battery-vf-Affective score			0.50 ****	0.39 ****	0.58 ****	0.18 ****	0.15 ***	0.13 **	0.17 ***	−0.17 ***
ToM Battery-vf-Cognitive score				0.48 ****	0.55 ****	0.26 ****	0.22 ****	0.20 ****	0.14 *	−0.22 ****
ToM Battery-vf-Mixed score					0.42 ****	0.17 ****	17 ****	0.10 *	0.02	−0.13 *
ToMI1-vf									0.26 ****	−0.32 ****
ERC-vf-RE										−0.21 ****
ERC-vf-Dys										

Note. * *p* < 0.05; ** *p* < 0.01; *** *p* < 0.005; **** *p* < 0.001. ToM = Theory of Mind; ToMI1-vf = Theory of Mind Inventory 1–French version; GEM-vf: Griffith Empathy Measure; ERC-vf = French version of Emotion Regulation Checklist; RE = emotion regulation; Dys = emotion dysregulation.

**Table 7:** Correlations between scores in ToM Battery-vf and ToMI1-vf for ID children.

	1	2	3	4	5
1 ToM Battery-vf-Total		0.76 ***	0.80 ***	0.60 ***	0.38 **
2 ToM Battery-vf-Affective score			0.43 ***	0.26 **	0.22 *
3 ToM Battery-vf-Cognitive score				0.19 **	0.33 *
4 ToM Battery-vf-Mixed score					0.30 *
5 ToMI1-vf					

Notes. * *p* < 0.05; ** *p* < 0.01; *** *p* < 0.001; ID = intellectually disabled.

## Data Availability

The data presented in this study are available on request from the corresponding author. The data are not publicly available due to privacy and ethical restrictions.
